# Vitamin B12 in Leber hereditary optic neuropathy mutation carriers: a prospective cohort study

**DOI:** 10.1186/s13023-022-02453-z

**Published:** 2022-08-09

**Authors:** Julia Zibold, Bettina von Livonius, Hana Kolarova, Günter Rudolph, Claudia S. Priglinger, Thomas Klopstock, Claudia B. Catarino

**Affiliations:** 1 Department of Neurology, Friedrich-Baur Institute, University Hospital, Ludwig-Maximilian University (LMU) Munich, Ziemssenstr. 1a, 80336 Munich, Germany; 2 Department of Ophthalmology, University Hospital, Ludwig-Maximilian University (LMU) Munich, Munich, Germany; 3 Department of Pediatrics and Inherited Metabolic Disorders, First Faculty of Medicine, Charles University and General University Hospital, Prague, Czech Republic; 4 German Center for Neurodegenerative Diseases (DZNE), Munich, Germany; 5 Munich Cluster for Systems Neurology (SyNergy), Munich, Germany

**Keywords:** Cobalamin, Vitamin B12, Hereditary optic atrophy, Leber optic atrophy, LHON, Mitochondrial disease

## Abstract

**Background:**

Leber hereditary optic neuropathy (LHON) is the most common mitochondrial disorder, frequently resulting in acute or subacute severe bilateral central vision loss. Vitamin B12 deficiency is also a known cause of optic neuropathy through mitochondrial dysfunction. Here we evaluated the prevalence and clinical significance of vitamin B12 deficiency in a large cohort of LHON patients and asymptomatic mutation carriers from a tertiary referral center.

**Methods:**

From the Munich LHON prospective cohort study, participants included all LHON patients and asymptomatic LHON mutation carriers, who were recruited between February 2014 and March 2020 and consented to participate. Neurological, general, and ophthalmological examinations were regularly performed, as were laboratory tests. Vitamin B12 deficiency was diagnosed if serum vitamin B12 was below 201 pg/mL, or if 201–339 pg/mL plus low serum holotranscobalamin or elevated serum methylmalonic acid or elevated total plasma homocysteine.

**Results:**

We analyzed 244 subjects, including 147 symptomatic LHON patients (74% males) and 97 asymptomatic mutation carriers (31% males). Median age at study baseline was 34 years (range 5–82 years). The prevalence of vitamin B12 deficiency was higher for LHON mutation carriers than for the general population in all age categories. This was statistically significant for the LHON mutation carriers under 65 years (21% vs. 5–7%, *p* = 0.002). While vitamin B12 deficiency prevalence was not statistically different between LHON patients and asymptomatic mutation carriers, its clinical correlates, e.g., macrocytosis and polyneuropathy, were more frequent in the subgroup of LHON patients. Excessive alcohol consumption was a significant predictor of vitamin B12 deficiency (*p* < 0.05).

**Conclusions:**

The high prevalence of vitamin B12 deficiency in LHON mutation carriers, both asymptomatic mutation carriers and LHON patients, highlights the need for regular vitamin B12 screening in this population, in order to ensure early treatment, aiming for better outcomes. Our study is not conclusive regarding vitamin B12 deficiency as determinant for disease conversion in LHON, and further research is warranted to disentangle the role of vitamin B12 in the pathophysiology and prognosis of LHON.

## Background

Leber hereditary optic neuropathy (LHON, OMIM 535000) is the most common primary mitochondrial disorder, with an estimated prevalence of 1 in 30,000 [1]. LHON frequently results in severe bilateral central vision loss [2], and the visual prognosis is often poor [3]. The onset of visual loss most commonly occurs in young patients between the second and fourth decades of life [4], but it can also develop in children or the elderly [5, 6].

LHON is usually caused by one of three primary mitochondrial DNA (mtDNA) point mutations, m.11778G > A in the MT-*ND4* gene, m.3460G > A in MT-*ND1*, or m.14484T > C in MT-*ND6* [7–9]. These genes encode subunits of complex I of the mitochondrial respiratory chain. LHON is mostly maternally inherited and shows incomplete penetrance [10]. There is a significant gender bias, with male LHON mutation carriers more likely to develop symptoms (male-to-female ratio 3:1–8:1) [4]. The clinical symptoms in LHON are due to mitochondrial dysfunction of the retinal ganglion cells (RGC). Impaired adenosine triphosphate (ATP) synthesis by oxidative phosphorylation (OXPHOS) and increased production of reactive oxygen species (ROS) lead to dysfunction and apoptosis of a proportion of the RGCs [2, 11].

The mechanisms underlying disease conversion from asymptomatic mutation carriers to manifest LHON disease are not fully understood. Both genetic and environmental factors modulate the risk of LHON disease [2, 4]. Smoking increases LHON conversion risk, as does excessive alcohol consumption [3]. Medications such as antimicrobials or antiepileptic drugs, or head trauma, may precede onset of visual loss in LHON [2, 12, 13]. Anecdotal evidence suggests that vitamin B12 deficiency may trigger vision loss in LHON mutation carriers [14, 15].

Vitamin B12 (B12, cobalamin) is essential for DNA synthesis [16] and requires regular intake as it is not synthesized in the human body [17]. It is a cofactor for two essential enzymatic reactions: one located in the mitochondria, where 5-deoxyadenosylcobalamin is necessary for methylmalonyl-coenzyme A (CoA) mutase to mediate the conversion of methylmalonyl-CoA to succinyl-CoA [18]; and the other in the cytosol, where methionine synthase depends on both 5-methylcobalamin and 5-methyltetrahydrofolate to catalyze the conversion of homocysteine to methionine, needed for the synthesis of proteins and the methyl donor S-adenosylmethionine [17]. Consequently, both methylmalonic acid (MMA) and total plasma homocysteine (tHCy) are elevated in vitamin B12 deficiency and may be used as biomarkers [17]. Similarly, low levels of holotranscobalamin (holoTC), the bioactive protein-bound form of B12, may signify B12 deficiency [19]. The metabolism of folate overlaps these pathways. Importantly, folate stores deplete faster than those of B12, and folate deficiency may lead to elevated tHCy even in the absence of B12 deficiency [20].

Vitamin B12 deficiency is relatively common in the general population worldwide [21]. Its estimated prevalence is dependent on age. The prevalence of B12 deficiency in Germany was estimated at 5–7% for people younger than 65 years, and at 10–30% in people 65 years and older [22].

There is no consensus in literature and clinical practice on a uniform definition for B12 deficiency, nor on the biomarkers and cut-off values to be used [17, 22–27]. Extended B12 diagnosis using other serum biomarkers has been shown to have higher sensitivity compared to measuring serum B12 level alone [28]. Using two or more of these biomarkers is more reliable [18, 29]. Low serum B12 levels can indicate B12 deficiency, but are not necessary for the diagnosis, as even low-normal or marginal serum B12 levels may indicate B12 deficiency when in combination with another abnormal biomarker [30, 31].

B12 deficiency can lead to hematological abnormalities (e.g., macrocytic anemia, pancytopenia), neuropsychiatric disorders, including memory impairment or depression, or even neurological deficits, including paresthesias, peripheral neuropathy, or spastic paraparesis [27, 32, 33]. It may also increase risk of cardiovascular disease through hyperhomocysteinemia [34, 35].

Importantly, B12 deficiency may lead to nutritional optic neuropathy resulting in loss of central vision, through mitochondrial dysfunction and impaired oxidative metabolism [36]. Thus, optic neuropathy due to vitamin B12 deficiency has common pathophysiologic features and similar clinical presentation to LHON.

Early diagnosis of B12 deficiency may allow effective treatment of any symptoms [27, 37] and prevent an irreversible condition [22]. Screening for B12 deficiency is therefore recommended for groups at risk, including older age, reduced intake of animal food, chronic alcohol abuse, or gastrointestinal disorders [22, 27]. Treatment is equally effective with oral or intramuscular B12 supplementation [38].

To date, no study has systematically analyzed the impact of B12 deficiency in LHON and to what extent it may influence the risk of disease conversion in asymptomatic mutation carriers.

This study aims to investigate the prevalence of B12 deficiency in LHON mutation carriers compared to the general population, to identify determinants of B12 deficiency in the LHON cohort, and to examine whether vitamin B12 affects the prognosis of LHON disease.

## Methods

### Participants; LHON patients and asymptomatic LHON mutation carriers

We included all LHON mutation carriers seen consecutively from February 2014 to March 2020 in the prospective LHON cohort study. All participants and legal guardians (for participants under the age of 18) gave written informed consent or assent. The local Ethics committee of the Ludwig-Maximilians-University (LMU) of Munich approved this project (project number 278-13).

### Study design

Participants are examined at baseline and followed up thereafter at regular intervals in the outpatient departments of Neurology and Ophthalmology. LHON patients were examined yearly, and asymptomatic mutation carriers at least every 2 years. Visits to the clinic included a complete medical history, with detailed history on comorbidities, medications, smoking and alcohol consumption habits. A systematic questionnaire on dietary habits was not conducted. General, neurological, and ophthalmological examinations were performed at each visit.

### Visual acuity assessments

Best-corrected visual acuity was measured using the Early Treatment Diabetic Retinopathy Study (ETDRS) chart. Clinically relevant recovery was defined as in previous LHON clinical studies [39, 40] as improvement in at least one eye in the logarithm of the Minimum Angle of Resolution (logMAR) of at least − 0.2, or from “off-chart” to “on-chart”.

### Excessive alcohol consumption, medications, and comorbidities

The weekly alcohol consumption was recorded, and an average daily amount was estimated. Excessive alcohol consumption was defined as exceeding 24 g of alcohol per day for men and 12 g per day for women [41].

All medications and comorbidities were reviewed for possible impact on serum B12 levels and B12 biomarkers. Medications potentially affecting serum B12 levels include metformin, proton pump inhibitors, histamine 2 receptor antagonists, chronic inhalation of nitrous oxide, highly active antiretroviral therapy, and oral contraceptives [17, 42].

### Laboratory measurements

We measured serum B12 and the additional B12 biomarkers MMA, tHCy, and holoTC at each visit. Likewise, serum folate and routine laboratory tests, including complete blood count and serum creatinine, were examined. We checked for laboratory abnormalities found in B12 deficiency: macrocytosis, macrocytic anemia, pancytopenia [32]. Subjects were excluded from the final analysis when they had increased serum creatinine indicating renal insufficiency, and also when they had folate deficiency with serum B12 higher than 201 pg/mL.

### Definition of vitamin B12 deficiency

Low serum B12 level was defined as < 201 pg/mL (< 148 pmol/L), and low-normal (marginal) serum B12 level between 201 and 339 pg/mL (148–250 pmol/L). Cut-offs for the other B12 biomarkers were set according to the validated reference values in our laboratory: reduced serum holoTC < 40 pmol/L, increased serum MMA > 300 nmol/L, and increased plasma tHCy > 12.0 µmol/L.

**Fig. 1:**
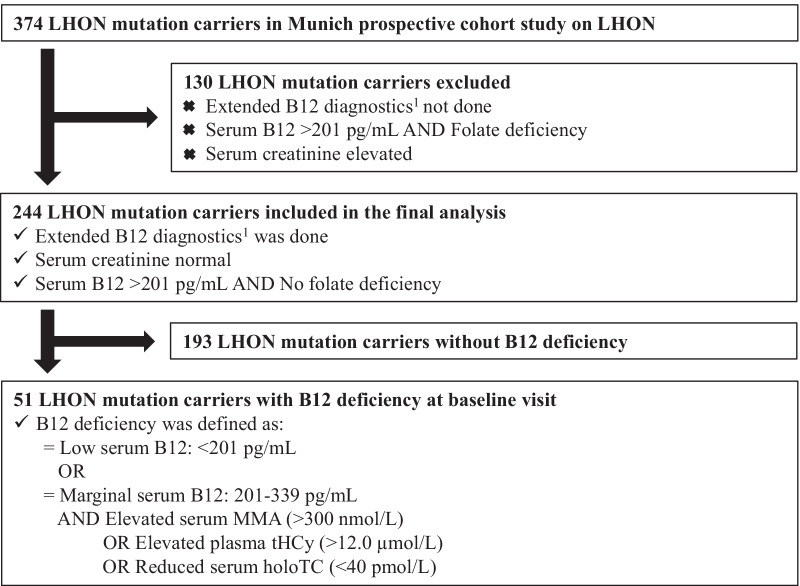
Flowchart of study inclusion and exclusion criteria

There is no universal guideline, and the definition of B12 deficiency used in this study was adapted from a current expert review on the recent recommendations [17]. A low serum B12 value by itself was sufficient for the diagnosis of B12 deficiency. If a low-normal (marginal) B12 level was present, B12 deficiency was diagnosed only if one of three conditions occurred: low serum holoTC, or elevated serum MMA, or elevated plasma tHCy (Fig. 1). Subjects with abnormal B12 biomarker (low holoTC, or elevated MMA, or elevated tHCy) at normal serum B12 levels (> 339 pg/mL) were not classified as B12 deficient.

### Data analysis

The frequency of B12 deficiency was compared between LHON patients and asymptomatic LHON mutation carriers, and with the German general population. Data were stratified by age categories (< 20; 20–39; 40–59; and > 60 years) and by gender. Pearson chi-square and Fisher’s exact test were used for binominal data. Non-parametric dependent McNemar test was used for the analysis of categorical data. Logistic regression was performed to look for predictors of LHON disease status, with B12 deficiency, gender, age category, excessive alcohol consumption, and smoking included in the model. A *p* value below 0.05 was considered statistically significant. All statistical analysis was performed using SPSS version 26.0 for Windows (Armonk, NY: IBM Corp., 2018).

## Results

### Description of the LHON cohort

We included 147 (60%) LHON patients and 97 (40%) asymptomatic LHON mutation carriers in the final analysis (Table 1, Fig. 1). The median age at study baseline was 34 years (range 5–82 years). Male gender was significantly more prevalent among LHON patients than asymptomatic mutation carriers (74% males vs. 31%, *p* < 0.001). LHON mutations were distributed similarly to other cohorts in the literature (Table 1) [4].

**Table 1:** Characteristics of the study population of LHON mutation carriers at baseline visit

	Total LHON cohort	LHON patients	Asymptomatic LHON carriers
Total, n	244	147	97
Median age at baseline, y (range)	34 (5–82)	34 (5–82)	35 (6–70)
Median age at disease onset, y (range)	NA	22 (3–80)	NA
Median disease duration, y (range)	NA	3 (2wk-54y)	NA
Gender, n (%)			
Male	138 (56.6)	108 (73.5)	30 (30.9)
Female	106 (43.4)	39 (26.5)	67 (69.1)
Male/female ratio	1:0.8	1:0.4	1:2.2
Mutation type, n (%)			
m.11778G > A	170 (69.7)	97 (66.0)	73 (75.3)
m.3460G > A	30 (12.3)	21 (14.3)	9 (9.3)
m.14484T > C	30 (12.3)	19 (12.9)	11 (11.3)
Others, rare LHON mutations	14 (5.7)	10 (6.8)	10 (4.1)
B12, pg/mL, mean ± SD (range)	440 ± 235 (150–2000)	446 ± 209 (150–2000)	432 ± 271 (150–2000)
HoloTC, pmol/L, mean ± SD (range)	73 ± 30 (13–132)	74 ± 31 (13–132)	71 ± 27 (15–128)
MMA, nmol/L, mean ± SD (range)	186 ± 103 (58–990)	186 ± 30 (72–735)	184 ± 117 (58–990)
tHCy, µmol/L, mean ± SD (range)	13 ± 6 (4–51)	14 ± 7 (4–51)	12 ± 4 (4–26)

*B12* vitamin B12, *holoTC* holotranscobalamin, *LHON* Leber hereditary optic neuropathy, *MMA* methylmalonic acid, *NA* not applicable, *SD* standard deviation, *tHCy* total homocysteine, *wk* weeks, *y* years

Among LHON patients, the median age at onset of visual loss for the first affected eye was 22 years (range 3–80 years). Idebenone, the only approved therapy to date [43], was taken by 79% of LHON patients. Clinically relevant recovery was documented in 39% of all LHON patients.

As previously described in our LHON cohort [44], the frequency of ever-smokers was significantly higher than in the general population (49% vs. 11%, *p* < 0.001) [45]. LHON mutation carriers also had significantly more excessive alcohol consumption than the general population (15% vs. 3.4%, *p* = 0.006) [45]. Compared to asymptomatic mutation carriers, LHON patients were both more likely to have ever smoked (*p* < 0.05) and to have excessive alcohol consumption (*p* < 0.001; Table 2a). LHON mutation carriers had moderately more polyneuropathy than the general population (13% vs. 5–8%) [46], more frequently among LHON patients than in asymptomatic mutation carriers (*p* = 0.003).

**Table 2:** Information on lifestyle factors and medical data related to B12 in the study population

	Total n = 244	LHON patients n = 147	Asymptomatic LHON carriers n = 97	*p* value
	n (%^#^)	n (%^#^)	n (%^#^)	
*(a) Total LHON cohort, stratified by LHON disease status*				
Smoking ever	118 (48.6)	79 (54.1)	39 (40.2)	0.034*
Excessive alcohol consumption ever	35 (14.5)	31 (21.4)	4 (4.2)	< 0.001***
Medications affecting B12	36 (15.2)	27 (18.8)	9 (9.7)	n.s
Comorbidities affecting B12	10 (4.1)	9 (6.2)	1(1.0)	n.s
Previous B12 supplementation	19 (11.2)	16 (16.8)	3 (4.1)	0.009**
Lab abnormalities related to B12	73 (32.6)	56 (41.2)	17 (19.3)	0.001**
Polyneuropathy	30 (12.7)	26 (17.8)	4 (4.6)	0.003**

	Total n = 51	LHON patients n = 30	Asymptomatic LHON carriers n = 21	*p* value
	n (%^#^)	n (%^#^)	n (%^#^)	
*(b) LHON cohort with B12 deficiency, stratified by LHON disease status*				
Smoking ever	31 (60.8)	21 (70.0)	10 (47.6)	n.s
Excessive alcohol consumption ever	12 (24.0)	11 (37.9)	1 (4.8)	0.007**
Medications affecting B12	10 (20.8)	6 (21.4)	4 (20.0)	n.s
Comorbidities affecting B12	2 (3.9)	2 (6.7)	0 (0.0)	n.s
Previous B12 supplementation	5 (21.7)	4 (28.6)	1 (11.1)	n.s
Lab abnormalities related to B12	18 (38.3)	14 (48.3)	4 (22.2)	n.s
Polyneuropathy	8 (16.3)	7 (23.3)	1 (5.3)	n.s

B12 deficiency	Yes n = 51	No n = 193	*p* value
	n (%^#^)	n (%^#^)	
*(c) Total LHON cohort, stratified by presence of B12 deficiency*			
Smoking ever	31 (60.8)	87 (45.3)	0.049*
Excessive alcohol consumption ever	12 (24.0)	23 (12.0)	0.033*
Medications affecting B12	10 (20.8)	26 (13.8)	n.s
Comorbidities affecting B12	2 (3.9)	8 (4.2)	n.s
Previous B12 supplementation	5 (21.7)	14 (9.6)	n.s
Lab abnormalities related to B12	18 (38.3)	73 (41.2)	n.s
Polyneuropathy	8 (16.7)	22 (11.6)	n.s

*B12* vitamin B12, *LHON* Leber hereditary optic neuropathy, *n.s.* not statistically significant

*p < 0.05, **p < 0.01, ***p < 0.001 (Pearson chi-square)

^#^Percentage values refer to the total number of data records available in each case

**Fig. 2:**
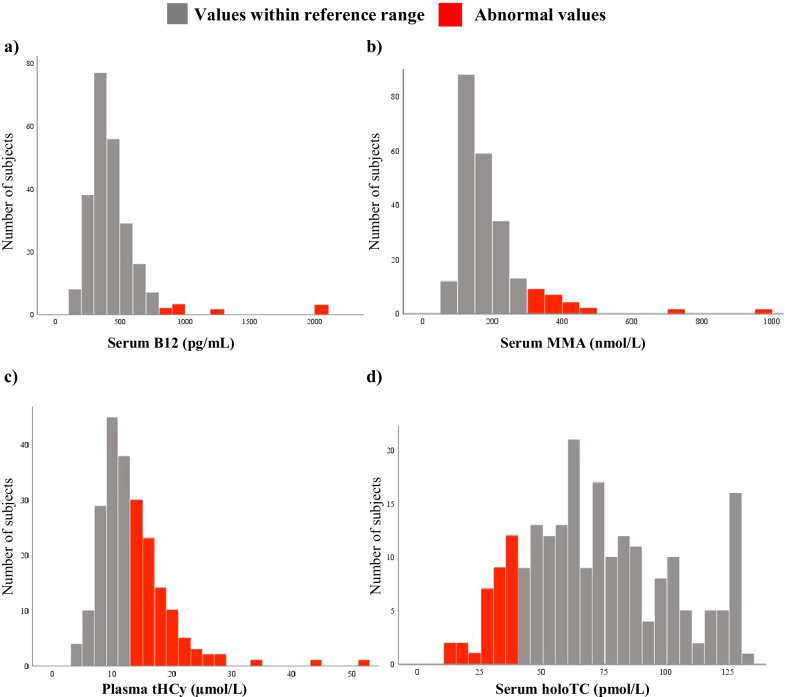
Distribution of the extended B12 diagnostic laboratory values in the LHON cohort. **a** serum vitamin B12 (B12); **b** serum methylmalonic acid (MMA); **c** total plasma homocysteine (tHCy); and **d** serum holotranscobalamin (holoTC)

**Fig. 3:**
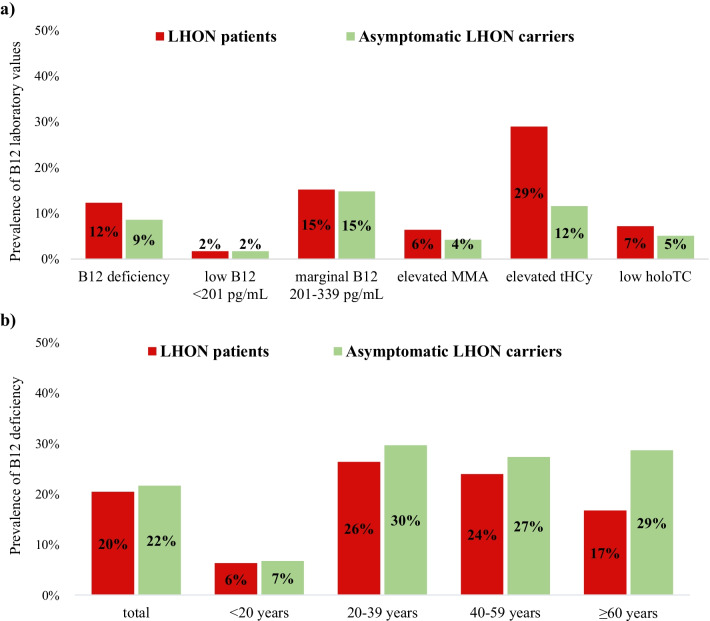
Prevalence of **a** abnormal B12 laboratory values and **b** B12 deficiency in the LHON cohort

The distribution of laboratory values for B12 deficiency biomarkers in the LHON cohort is shown in Table 1, Figs. 2a–d and 3a.

### Vitamin B12 deficiency at study baseline

At baseline, 21% (51/244) of all LHON mutation carriers had B12 deficiency. The prevalence of B12 deficiency varied among age categories in the LHON cohort (*p* < 0.05) and was most frequent between 20 and 59 years (25–27%; Table 3a, Fig. 3b). In comparison, B12 deficiency in the general population in Germany is 5–7% for those under 65 years of age and 10–30% for 65 years and older [22]. LHON mutation carriers in our cohort had a higher prevalence of B12 deficiency than the age-matched general population, statistically significant for under 65 years (*p* = 0.002; Table 3b).

**Table 3:** Prevalence of B12 deficiency stratified by age groups

	Total LHON cohort	LHON patients	Asymptomatic LHON carriers	*p* value
	n/N (%)	n/N (%)	n/N (%)	
*(a) B12 deficiency in LHON cohort*				
< 20 y	4/62 (6.5)	2/32 (6.3)	2/30 (6.7)	n.s
20–39 y	23/84 (27.4)	15/57 (26.3)	8/27 (29.6)	n.s
40–59 y	20/79 (25.3)	11/46 (23.9)	9/33 (27.3)	n.s
≥ 60 y	4/19 (21.1)	2/12 (16.7)	2/7 (28.6)	n.s
Total	51/244 (20.9)	30/147 (20.4)	21/97 (21.6)	n.s

	Total LHON cohort	German population [22]	*p* value
	n/N (%)	n/N (%)	
*(b) B12 deficiency in LHON cohort compared to the general population*			
< 65 y	48/234 (20.5)	(5–7)	0.002**
≥ 65 y	3/10 (30)	(10–30)	n.s

*B12* vitamin B12, *LHON* Leber hereditary optic neuropathy, *n.s.* not statistically significant, *y* years

**p < 0.01 (Pearson chi-square)

There was no difference in the prevalence of B12 deficiency between genders. Importantly, we did not find a significant difference between LHON patients and asymptomatic mutation carriers (20% vs. 22%, respectively), irrespective of gender and age group (Table 3a, Fig. 3b). However, significantly more LHON patients reported having taken B12 compared to asymptomatic mutation carriers (*p* = 0.009; Table 2a). Previous B12 supplementation was reported in 22% of all LHON mutation carriers who were B12 deficient at baseline.

We found significantly more smokers and subjects with excessive alcohol consumption among the LHON subgroup with B12 deficiency compared to individuals without (*p* < 0.05, respectively; Table 2c). Excessive alcohol consumption stated significantly more LHON patients than asymptomatic mutation carriers (*p* = 0.007). Within the B12-deficient subgroup of the LHON cohort, 38% had hematological abnormalities such as macrocytosis and 16% had polyneuropathy (Table 2c).

A total of 8% of subjects had both B12 deficiency (with low serum B12 < 201 pg/mL and at least one abnormal B12 biomarker) and concomitant low serum folate levels. These subjects had neither excessive alcohol consumption nor polyneuropathy.

A clinically relevant recovery (CRR) at the last follow-up was documented for 30% of LHON patients with B12 deficiency at baseline, compared to 42% of LHON patients without B12 deficiency.

Having medications or comorbidities known to modulate serum B12 levels did not affect whether LHON mutation carriers were B12 deficient (Table 2c).

Finally, in a multivariate analysis, we confirmed gender (*p* < 0.001) and excessive alcohol consumption (*p* = 0.004) to be significant predictors of LHON disease status, which is in line with the literature [4]. Interestingly, B12 deficiency was not a significant predictor of LHON disease status. Only excessive alcohol consumption was found to be a significant predictor of B12 deficiency (*p* = 0.027).

### B12 deficiency at follow-up visits

The prevalence of B12 deficiency for LHON mutation carriers did not significantly decrease at the follow-up (FUP) visits compared to study baseline, despite diagnosis and recommendations for treatment. Surprisingly, in the LHON subgroup with B12 deficiency at baseline, 42% continued to be B12 deficient at FUP 1; 50% at FUP 2; and 67% at FUP 5. In addition, 19% of LHON mutation carriers without B12 deficiency at baseline later presented with B12 deficiency at FUP 1.

### Variation of B12 deficiency findings by consideration of serum B12 levels or biomarkers

Using extended biomarkers detects significantly more B12 deficiency than measuring serum B12 alone (21% vs. 3%, *p* < 0.001), especially in the age group 20–59 years (*p* < 0.001). Among the LHON mutation carriers who fulfilled the criteria for B12 deficiency, 24% had elevated MMA, 73% had elevated tHCy, and 30% had low holoTC. Low-normal B12 levels were more frequent in asymptomatic mutation carriers than in LHON patients (*p* < 0.05). Hyperhomocysteinemia occurred more frequently in LHON patients than in asymptomatic mutation carriers (*p* = 0.002).

## Discussion

In our large, well-characterized cohort of LHON mutation carriers, we could show that both LHON patients and asymptomatic LHON mutation carriers have a higher prevalence of B12 deficiency compared to an age-matched general population. This was statistically significant among LHON mutation carriers younger than 65 years. Interestingly, it was most pronounced in the age group 20–39 years, where the onset of visual loss in LHON is most frequent [4].

The sample size for LHON mutation carriers aged 65 years and older is too small to allow a robust comparison with an age-matched general population. Although the LHON cohort overall has a more limited age range than the general population, we could control for this by performing age-matched comparisons.

We excluded any subject without laboratory data on B12 or biomarkers from the final analysis. As the group of excluded subjects was not significantly different from that of included individuals for all relevant demographic and clinical variables, there was no systematic selection bias.

A high proportion of LHON mutation carriers with B12 deficiency presented related clinical features, most frequently hematological abnormalities such as macrocytosis, or polyneuropathy. This finding helps to highlight the clinical impact of consistently screening for B12 deficiency in the LHON cohort, as this is a treatable disorder when diagnosed early and can lead to symptomatic disease if left untreated. We excluded folate deficiency as the underlying cause of macrocytosis. Of note, some subjects with B12 deficiency and polyneuropathy also had a history of excessive alcohol consumption, which may also have contributed to polyneuropathy due to toxicity and poor nutrition.

The additional presence of symptoms, such as peripheral neuropathy, in LHON patients is sometimes referred to as LHON plus disease [2]. A proportion of what is currently considered LHON plus may actually be due to comorbid B12 deficiency causing peripheral neuropathy or spastic paraparesis.

We accounted for comorbidities and medications potentially affecting serum B12 levels [17]. For example, oral contraceptives may lead to falsely low serum B12 levels [42]. Comorbidities affecting serum B12 levels include atrophic gastritis, gastrectomy, ileal resection, bariatric surgery, and inflammatory bowel diseases [23]. Hyperhomocysteinemia may be caused by isolated folate deficiency, vitamin B6 deficiency, renal insufficiency, hypothyroidism, or genetic variants [26]. MMA is elevated in patients with renal dysfunction, reduced gastric acidity, and intestinal bacterial overgrowth [17]. HoloTC is reduced in renal insufficiency or liver disease [17, 30].

We found higher rates of previous B12 supplementation in LHON patients. Nevertheless, there was a significant amount of missing data on B12 supplementation for our cohort, which may have led to underestimation of the true prevalence of B12 deficiency. Self-reporting of vitamin supplementation and medication intake, as well as alcohol and tobacco consumption, may have created some reporting bias. Also, we determined the B12 status of LHON patients at study baseline, and there was a lag of 3 years (median) between onset of symptoms and study baseline. This is a limitation of the study design, as vitamin B12 deficiency immediately before onset of vision loss would be more relevant for the risk of disease conversion.

We found a high prevalence of smoking and excessive alcohol consumption in our cohort, especially among LHON patients. Both conditions were significantly more frequent in the subgroup of LHON patients with B12 deficiency. Patients with excessive alcohol consumption are known to represent a high-risk group for vitamin deficiency, including B12 deficiency [47]. Using multivariate analysis, we confirmed that excessive alcohol consumption is a significant predictor for LHON disease status and for B12 deficiency.

Nevertheless, we may have underestimated the true prevalence of B12 deficiency in the LHON subgroup with excessive alcohol consumption: Not only serum B12 may be falsely elevated in subjects with excessive alcohol consumption [47–50], but also they present with hyperhomocysteinemia [47]. It is unclear whether subjects with excessive alcohol consumption, normal serum B12, and increased tHCy have a true vitamin B12 deficiency. The definition of B12 deficiency we used includes subjects with elevated tHCy only when there is concomitantly a low-normal serum B12 (≤ 339 pg/mL).

More than a quarter of our LHON cohort had hyperhomocysteinemia, and still was not classified as having B12 deficiency. Some of these individuals may have been misclassified as not having vitamin B12 deficiency due to falsely elevated B12 levels, which are known to occur with excessive alcohol consumption.

Importantly, elevated tHCy levels are associated with increased risk of neuronal damage [16, 27], neurodegenerative diseases such as dementia [27, 30], and thromboembolic events [51, 52]. Nearly half of the LHON cohort had hyperhomocysteinemia. Together with elevated MMA, it may be relevant as a trigger and determinant for the progression of neurological diseases [18]. This highlights the need for an extended B12 diagnosis in LHON mutation carriers to have enough sensitivity to correctly rule out or diagnose vitamin B12 deficiency. In addition, it demonstrates the need to inform all LHON mutation carriers on the importance of avoiding excessive alcohol consumption.

While our study is inconclusive on B12 deficiency and risk of disease conversion in asymptomatic LHON mutation carriers, further research is warranted.

### Vitamin B12 deficiency and the final pathway of mitochondrial dysfunction

The papillomacular bundle of the retinal ganglion cells (RGC) is vulnerable to mitochondrial dysfunction and affected early by it [2, 53]. Carrying a LHON mutation already implies a genetic susceptibility to mitochondrial dysfunction leading to LHON disease, while having a B12 deficiency may lead on its own to mitochondrial impairment and nutritional optic neuropathy [36]. B12 is an efficient free radical trap, and B12 deficiency disrupts oxidative metabolism [16, 36]. Accumulation of its metabolites tHCy and MMA may also disturb mitochondrial function [16]. In addition, toxic causes of mitochondrial dysfunction, such as smoking, lead to an increased risk of optic neuropathy [36]. Effectively, the combination of genetic risk in LHON mutation carriers with the nutritional risk (B12 deficiency), and toxic risk (smoking, excessive alcohol consumption) may increase the risk of conversion from asymptomatic to the final pathway of mitochondrial dysfunction leading to optic neuropathy. Impaired OXPHOS causes less ATP and subsequently more ROS formation due to oxidative stress, which results in increased dysfunction and apoptosis of RGC, and therefore optic neuropathy [36]. Our study shows that LHON mutation carriers have a high prevalence of B12 deficiency, although the exact mechanism is still unclear. It remains unclear whether B12 deficiency may trigger manifestation of the disease in LHON mutation carriers. Importantly, B12 deficiency is an easily diagnosed and treatable condition that may contribute to mitochondrial dysfunction, for which LHON mutation carriers are predisposed.

B12 supplementation may benefit selected individuals with neurological disorders [27]. However, it was not yet systematically evaluated in patients with mitochondrial diseases [54, 55]. Current treatment recommendation for LHON patients in the first years after symptom onset is idebenone [43], which optimizes ATP production and is a ROS scavenger [36]. LHON patients often take off-the-counter multivitamin supplementation, which has currently insufficient evidence [2], apart from case reports [14, 15]. Treatment of other combined metabolic optic neuropathies such as tobacco-alcohol amblyopia, consists of abstinence from alcohol and tobacco, and vitamin supplementation may also help improve prognosis [36]. All of this highlights the need for longer-term prospective studies of systematic B12 supplementation in LHON mutation carriers and its effects on the rate of both disease conversion and clinically relevant recovery.

Our study demonstrated that LHON mutation carriers are a population at risk for B12 deficiency. They need to be informed about screening for B12 deficiency and its treatment at each visit. For all subjects with B12 deficiency, we recommend supplementation with oral or intramuscular B12, followed by repeat B12 measurements after 2–3 months. However, many patients with B12 deficiency continued to have a low B12 status at subsequent follow-up visits. We consider the following reasons: non-compliance with vitamin supplementation despite treatment recommendations unless serum B12 levels were below 201 pg/mL; unawareness of B12 deficiency; refractoriness to B12 supplementation; or unremarkable B12 values between visits. Inadequate treatment caused preventable neurological conditions such as polyneuropathy in some cases. One subject even presented with memory deficits and spastic paraparesis. All of this emphasizes the need for better awareness among physicians and patients about the diagnostic value of the more sensitive B12 biomarkers. Furthermore, regular monitoring in primary care is essential.

### Definition of B12 deficiency and use of B12 biomarkers

A universally accepted definition of B12 deficiency, including the biomarkers to be used in clinical practice, remains controversial. Depending on the definition, the true prevalence of B12 deficiency may differ. One study found a specificity of only 60% in subjects with B12 levels below 200 pg/mL [56]. We chose a definition of B12 deficiency adapted from recent literature, combining serum B12 levels with the biomarkers holoTC, MMA, and tHCy. In addition, we systematically performed a thorough medical history and examination, measured serum creatinine and serum folate. Thus, we improved the sensitivity and specificity for assessing the B12 status in our LHON cohort. However, the use of all four B12 biomarkers is costly [17], making our approach challenging to apply in primary practice. Although some recommend screening for B12 deficiency by examining holoTC first [19], each biomarker alone has insufficient sensitivity and specificity to determine B12 deficiency correctly [17, 18, 57]. Measuring at least total plasma homocysteine in addition to serum B12 may be a cost-effective option to increase the yield of diagnosis of B12 deficiency in clinical practice. Further, we found a large proportion of subjects with unexplained polyneuropathy or macrocytosis. At least in part, this may be due to misclassification of B12 deficiency. In all cases, it is unclear whether these individuals could have benefited from B12 supplementation.

## Conclusions

Our study demonstrates a high prevalence of B12 deficiency in LHON mutation carriers.

While further prospective studies will be required for definite conclusions, our data suggests that screening for B12 deficiency may potentially be included in the LHON guidelines, as early detection and effective treatment of B12 deficiency may prevent neurological impairment. Longer-term prospective studies examining B12 deficiency and systematic, adequate B12 supplementation in LHON cohorts are warranted.

## Data Availability

Any data from this work not published within this article will be shared on reasonable request from any qualified investigator. Individual participant data will only be shared in a de-identified form.

## Abbreviations

ATP: Adenosine triphosphate
B12: Vitamin B12
CoA: Coenzyme A
ETDRS: Early treatment diabetic retinopathy study
FUP: Follow-up
holoTC: Holotranscobalamin
LHON: Leber hereditary optic neuropathy
logMAR: Logarithm of the minimum angle of resolution
MMA: Methylmalonic acid
mtDNA: Mitochondrial DNA
OXPHOS: Oxidative phosphorylation
RGC: Retinal ganglion cells
ROS: Reactive oxygen species
tHCy: Total plasma homocysteine
